# Health-related rehabilitation services: assessing the global supply of and need for human resources

**DOI:** 10.1186/1472-6963-11-276

**Published:** 2011-10-17

**Authors:** Neeru Gupta, Carla Castillo-Laborde, Michel D Landry

**Affiliations:** 1 Health Workforce Information and Governance, Department of Human Resources for Health, World Health Organization, Geneva, Switzerland; 2 Health Planning Division, Public Health Secretariat, Ministry of Health, Santiago, Chile; 3 Doctor of Physical Therapy Division, Department of Community and Family Medicine, Duke University, Durham, North Carolina, USA

## Abstract

**Background:**

Human resources for rehabilitation are often a neglected component of health services strengthening and health workforce development. This may be partly related to weaknesses in the available research and evidence to inform advocacy and programmatic strategies. The objective of this study was to quantitatively describe the global situation in terms of supply of and need for human resources for health-related rehabilitation services, as a basis for strategy development of the workforce in physical and rehabilitation medicine.

**Methods:**

Data for assessing supply of and need for rehabilitative personnel were extracted and analyzed from statistical databases maintained by the World Health Organization and other national and international health information sources. Standardized classifications were used to enhance cross-national comparability of findings.

**Results:**

Large differences were found across countries and regions between assessed need for services requiring health workers associated to physical and rehabilitation medicine against estimated supply of health personnel skilled in rehabilitation services. Despite greater need, low- and middle-income countries tended to report less availability of skilled health personnel, although the strength of the supply-need relationship varied across geographical and economic country groupings.

**Conclusion:**

The evidence base on human resources for health-related rehabilitation services remains fragmented, the result of limited availability and use of quality, comparable data and information within and across countries. This assessment offered the first global baseline, intended to catalyze further research that can be translated into evidence to support human resources for rehabilitation policy and practice.

## Background

An estimated one billion people worldwide experience some form of disability and are in need of health and rehabilitation services, the majority in low- and middle-income countries [1]. Despite the urgency of the issues at stake, prioritizing and monitoring of progress to improve health services for people with disabilities remains inadequate [2]. Notably, the Millennium Development Goals (MDGs) compact, meant to establish a unifying set of objectives on pressing health and development issues and encourage collaborative action among the global community, fails to explicitly mention rehabilitative health services or set service coverage targets for persons with disabilities [3].

Enhancing accessibility to health services means addressing the key constraints related to human resources for health (HRH). For one, efforts to implement the new and ambitious international guidelines for community-based rehabilitation (CBR) [4] are expected to place heavy demands on practitioners to work across disciplines and sectors to meet the medical and psychosocial needs of people with disabilities [5]. Yet despite their central role in services delivery, human resources for rehabilitation are an often neglected component of health systems development. Human resources for rehabilitation are often absent from national health sector plans and reviews or HRH development strategies [6].

Assessing the availability of rehabilitation health workers is a critical starting point for understanding the capacity of health systems to meet health-related rehabilitation service objectives in a country. A few studies have profiled the rehabilitation workforce and forecast gaps using different data sources and approaches, usually focusing on a single profession or practice modality and limited to a specific country or region [7-12]. Research on determinants of workforce supply and distribution among rehabilitation professionals is minimal [13].

One complexity in understanding the situation on human resources for health-related rehabilitation services is that there is no commonly adopted monitoring framework or universal "gold standard" for required human resources. In any health system, different categories of health workers may provide different forms of health and rehabilitation services. The specific mix of personnel needed in local contexts will vary depending on the circumstances of the area. For example, a country with large numbers of motor vehicle accidents may need more workers specialized to deal with cognitive and musculoskeletal impairments, whereas another country may need more workers skilled in providing services for disabilities associated with HIV/AIDS and other communicable diseases [1].

Not only do the settings for rehabilitation vary greatly from country to country, information on the availability of rehabilitation personnel to staff these settings is often only an estimate. Data remain fragmented and inadequate, especially in low- and middle-income countries. This is partly related to lack of common definitions and classifications, partly to poor availability and use of standard statistical sources for workforce monitoring, and partly to lack of political will to place monitoring of human resources for rehabilitation high on the health agenda - the latter itself may be related to the way societies often interpret and react to disability. For instance, Haig et al. facetiously concluded, taking into account the lack of documentation on physical and rehabilitation medicine in sub-Saharan Africa, the chance of a person with a disability in sub-Saharan Africa meeting a physician with specialist skills is about the same as that for an Antarctic penguin [14].

This study aims to strengthen the global information and evidence base on human resources for rehabilitation. Since the release by the World Health Organization (WHO) of its flagship publication on the health workforce, *The world health report 2006: working together for health *[15], an increasing number of studies have attempted to improve understanding empirically and methodologically of the global HRH situation in relation to selected health dimensions, notably those prioritized by the MDGs (see [16-18]); however, to the best of our knowledge none have examined the situation with regard to disability and rehabilitation. This article presents new cross-national findings of supply of and need for rehabilitative personnel within and across regions, as a basis for strategy development of the workforce in physical and rehabilitation medicine. It proposes a standardized approach for measuring and monitoring health workforce capacity to respond to population needs for rehabilitation services. The underlying objective is to encourage a greater number of countries and stakeholders to plan for an effective, sustainable rehabilitative health workforce and implement ongoing monitoring to inform decision-making for HRH policy and practice and enhanced accountability.

## Methods

Data on the supply of rehabilitative personnel are primarily drawn from a custom extract of statistical information on health occupations from official national sources collated in the WHO's *Global Atlas of the Health Workforce *[19], the main international database on health workforce information. Depending on the organization of national health systems and means of monitoring, the database captures information from various administrative sources including health facility staffing records, civil service payroll records and registries of health professional regulatory bodies, as well as from population-based sources such as censuses and surveys with questions on labour force activity and occupation. In order to enhance cross-national comparability of results, data were mapped to the latest revision of the International Standard Classification of Occupations (ISCO), known as ISCO-08, a hierarchical framework of titles and codes for classifying and aggregating occupational information according to similarities in skill level and skill specialization required to fulfil the tasks and duties of jobs [20].

We included the latest available data over the period 1991-2008, and focused on nine categories of personnel likely to be a vital part of teams working in rehabilitation health services (Table 1). While there is no single operational boundary of what constitutes the rehabilitation health workforce, the mapping of data and information to ISCO (or its national equivalent) provides a coherent framework for workforce categorization [21]. For example, although physiatrists (physicians with specialty training in physical medicine and rehabilitation) may have a wider repertoire of knowledge and diagnostic and therapeutic skills for persons needing rehabilitation, some physicians in general practice and family medicine have pragmatic knowledge of rehabilitation environments. Other types of personnel are also known to be essential to the provision of comprehensive health and rehabilitation services - health services managers, patient care assistants, community-based rehabilitation health workers, dieticians, orthopaedic shoemakers, wheelchair repairers, etc. - however, these were not included in our analysis given the paucity of available data.

**Table 1:** Occupations related to health and rehabilitation services mapped to the International Standard Classification of Occupations, 2008 revision

Occupational category	ISCO code*	Examples of national occupation titles
*Generalist medical practitioners*	2211	General medical practitioner, Family medical practitioner, Primary health care physician
*Specialist medical practitioners*	2212	Specialist physician (physical and rehabilitation medicine), Physiatrist, Orthopaedic surgeon
*Nursing professionals*	2221	Specialist nurse (physical therapy)
*Physiotherapists*	2264	Physiotherapist, Orthopaedic physical therapist
*Audiologists and speech therapists*	2266	Audiologist, Speech therapist, Speech-language pathologist
*Other health professionals*	2269	Occupational therapist
*Medical and dental prosthetic technicians*	3214	Orthotist, Orthotic technician, Prosthetist, Prosthetic technician, Orthopaedic appliance technician
*Physiotherapy technicians and assistants*	3255	Physiotherapy technician, Physiotherapy assistant, Rehabilitation technician, Massage therapy technician
*Other health associate professionals*	3259	Respiratory therapy technician

Source: Adapted from International Labour Organization [20]. *Note: Refers to the ISCO-08 code at the most disaggregated four-digit (unit group) level.

We further conducted web-based and bibliographic searches for additional data from official national sources on human resources supply published in health sector reviews, statistical bulletins and HRH strategic plans. We did not use data published in academic journals, books or other non-official sources. Some supplementary information was gathered from the website of an international non-governmental health professional association (World Confederation for Physical Therapy) of the voluntary associations among its member organizations [22]; follow-up electronic communications were sent directly to national correspondents for data validation, with a 52% response rate. All raw HRH data were translated into densities per 10,000 inhabitants in order to enable comparisons across populations and geographies.

Estimates of population need for rehabilitation services (albeit with a focus on medical need above other quality of life dimensions) were derived from the WHO *Global Burden of Disease *study [23], including data on cause-specific diseases, injuries and risk factors by country and region based on the best available evidence in 2008. Data from this source were mapped to the International Classification of Diseases to enhance comparability of findings [24]. Need was measured in terms of attributable years of life lost (YLL) as related to causes that are considered to require assistance of health professionals associated to rehabilitation. This included most types of non-communicable conditions and injuries as well as certain infectious diseases, maternal and perinatal conditions, and nutritional deficiencies. In the absence of an international standard for classifying health care procedures specific to rehabilitation, the determination was based on technical advice from WHO experts in disability and rehabilitation.

Datasets were merged and basic analyses were conducted using descriptive statistics and simple regression models to compare and contrast differences in terms of the two main variables -- that is, supply of and need for human resources for rehabilitation -- within and across countries and regions. Where appropriate, coefficients of determination were calculated to estimate the goodness of fit of the regression models.

## Results and discussion

### Supply of human resources for rehabilitation

**Figure 1:**
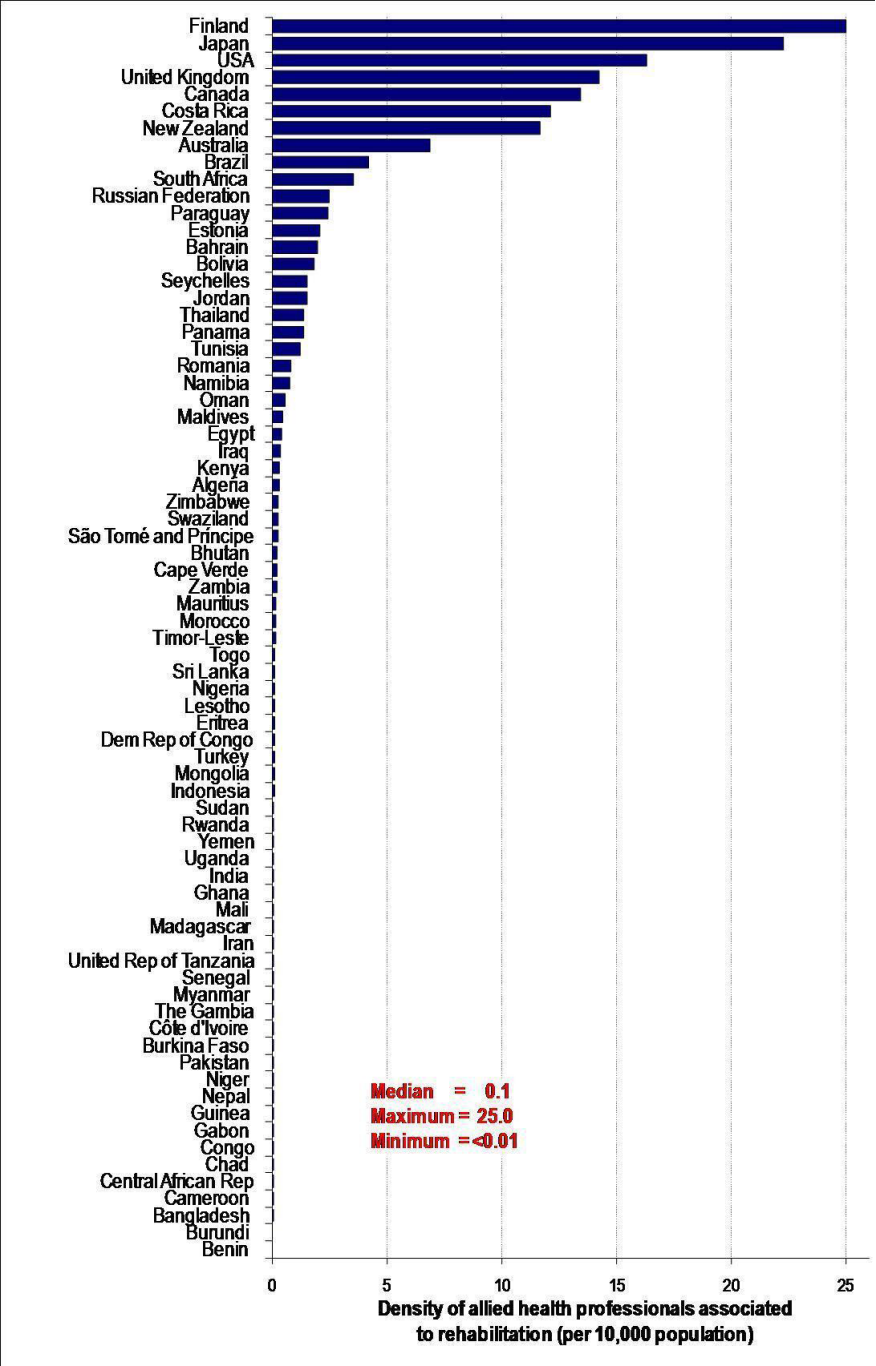
**Density of allied health workers associated to rehabilitation, 73 countries**. Source: Global Atlas of the Health Workforce [19].

Our investigation revealed wide cross-national disparities in the supply of allied health professionals associated to rehabilitation (except medical practitioners). Lower income countries tend to have the lowest densities: less than 0.5 workers per 10,000 inhabitants in many countries of sub-Saharan Africa (Burundi, Cameroon, Central African Republic, Chad, Congo, Gabon, Guinea, Niger, Burkina Faso, Cote d'Ivoire, Gambia, Senegal, Tanzania, Madagascar, Mali, Ghana, Uganda) but also in several across Asia (Bangladesh, Nepal, Pakistan, Myanmar, India) and the Eastern Mediterranean (Iran, Yemen). Many high income countries - including Finland, Japan, the United States, the United Kingdom and Canada - have workforce densities several times higher (Figure 1) [19]. This finding is not surprising: large differences across countries in overall HRH density and critical shortages of highly skilled professionals in low-income countries have been well documented internationally [15].

It is important to note that HRH data disaggregated for allied health occupations associated to rehabilitation were found for only 38% of WHO's 193 Member States. In the Americas, information was even scarcer: only 7 (20%) of the countries in the region had relevant data (Bolivia, Brazil, Canada, Costa Rica, Panama, Paraguay and the United States). Data coverage was higher in the African region, with 32 (70%) of 46 countries reporting statistics on rehabilitation personnel. This does not necessarily mean no data at all were available in other countries, but that data were not being collated and publicly disseminated through national government health or statistical channels and captured in the international database. Coverage for the African region may have been relatively high due to the results of a special data collection exercise conducted by WHO among health ministries and other partners to feed the empirical analysis of the *World health report 2006 *[25]. Coverage was surprisingly low for high income countries, but is expected to increase in coming years following expansion of a joint data collection exercise by the WHO European Regional Office and the Organization for Economic Cooperation and Development on health workforce statistics including more non-medical occupations [26].

Among countries with available data, differences were found in the number of occupations related to rehabilitation for which data were disseminated. South Africa had the largest number of categories at 16, counting those subject to national regulation and reported by the Health Professions Council of South Africa: medical orthotists and prosthetists, occupational therapists, occupational therapy technicians, orthopaedic footwear technicians, physiotherapists, speech therapists and others (results not shown). Elsewhere, in Bolivia and Costa Rica two types of allied rehabilitative personnel ("physiotherapists and related associate professionals" and "speech therapists") could be distinguished according to the harmonized occupational classification applied to the public use microdata release of the national population census of 2001 and of 2000, respectively. Likewise, only physiotherapists and speech pathologists were retained from the Australia 2001 census. In the United States, five types of therapists (occupational, physical, respiratory, speech and "other") were captured with the occupation variable of the internationally released Current Population Survey microdata file. For over half (44 or 60%) of the countries with available data, only information on numbers of physiotherapists (including sometimes related professions as per the applied occupational classification) was collected - e.g. for Benin, Cameroon, Egypt, Kenya, Iraq, Myanmar, Nigeria, Oman and Sri Lanka, among others.

**Figure 2:**
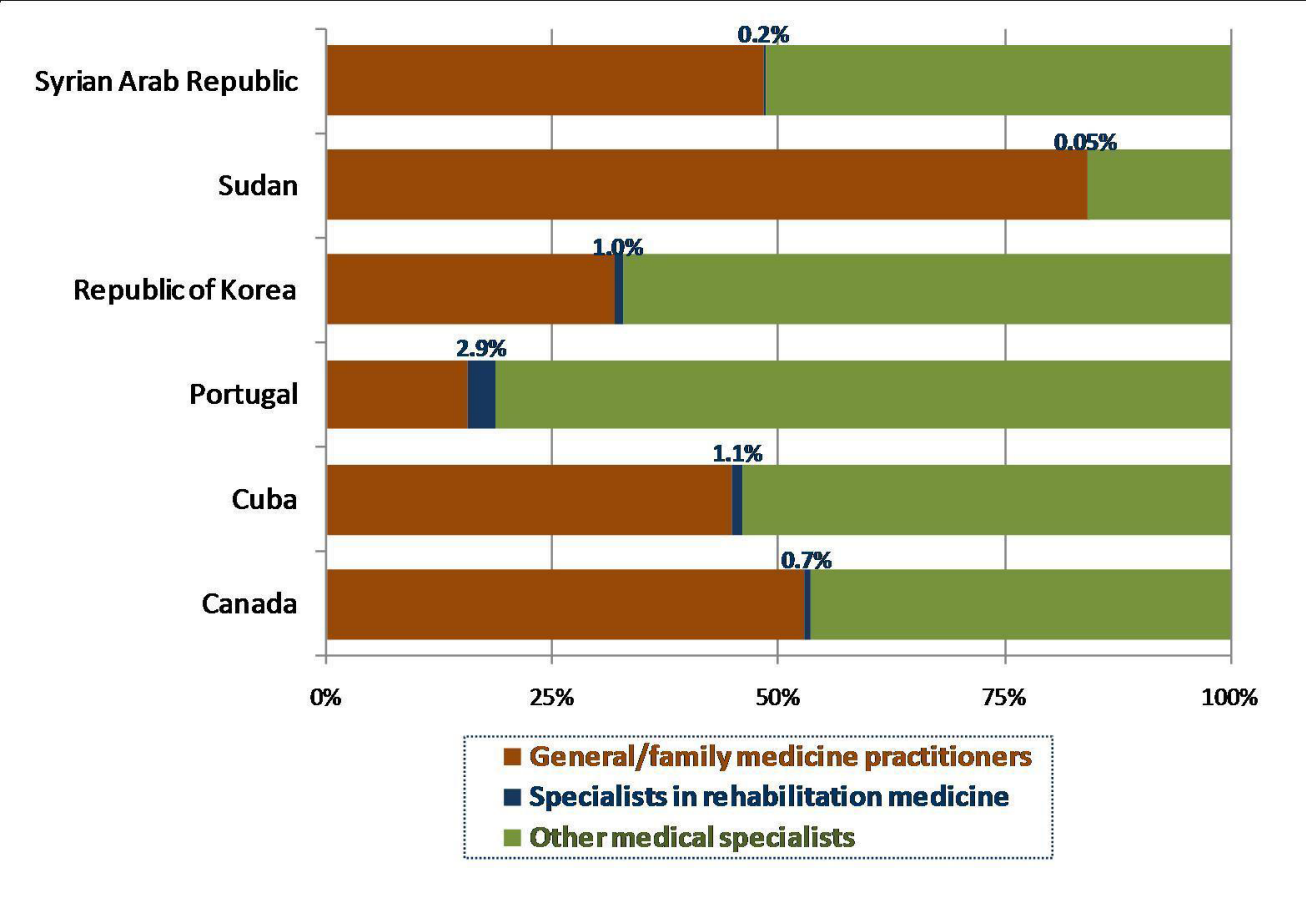
**Percentage distribution of the medical workforce by practice area, 6 countries**. Source: Canadian Institute for Health Information; Oficina Nacional de Estadísticas de Cuba; Alto Comissariado da Saude de Portugal; Statistics Korea; Sudan National Human Resources for Health Observatory; Syrian Central Bureau of Statistics [27-32].

Because the *Global Atlas *had very limited data on medical practice areas, we conducted further reviews of government publications to gather relevant information. Data from countries with published statistics on the distribution of the medical workforce teasing out specialists in physical and rehabilitation medicine are presented in Figure 2[27-32]. There is no global standard or norm for the minimum density of rehabilitation specialists or for their ratio to other categories of personnel [6], and this is reflected in the observed differences across countries. While in general the percent of the medical workforce specializing in rehabilitation medicine is low, less than 3% of all physicians, relatively large differences were found among the few countries with available data: the proportion was sixty times greater in Portugal than in Sudan, for example. This may be a reflection, in part, of the overall medical workforce distribution among generalist versus specialist practitioners, which is also subject to wide cross-national differences.

Given the variability in the nature of the underlying national information sources, comparability of the data remains uncertain, even under the application of a common occupational classification. Comparability may be hampered when it is not possible to ascertain whether the source of data covers health workers in all sectors (public facilities, private facilities, community-based service delivery, academic training, research, etc.) and types of activity (paid employment, self-employed, unemployed, retired...) [21]. For instance, occupation data from a population census usually cover individuals active in the national labour force over a given time period, as classified according to the nature of their main work activity, regardless of sector. Data from health professional regulatory bodies generally include individuals who have met certain qualifications and have registered with the appropriate body, regardless of current work activity or physical location in the country. Data from ministry of health administrative records oftentimes only cover public sector employees or posts.

An attempt was made to triangulate data from two different sources to better understand the potential differences in reporting. We compared data for physiotherapists, the profession with the largest number of data points, according to findings from official sources collated in the WHO's *Global Atlas *against those obtained from national professional associations. The latter are based on voluntary memberships, and so may either underestimate or overestimate actual supply of physiotherapists in a given country. For example, in Finland, both licensed physiotherapists and physiotherapy students may apply to become members of the Finnish Association of Physiotherapists [33], whereas official HRH statistics count all persons registered with the National Authority for Medicolegal Affairs [34] regardless of current practice. In Costa Rica, official data from the census refer to the main type of work in the week preceding enumeration [35] and may include professions performing similar types of rehabilitation work but with different professional titles, such as kinesiologists or ergotherapists, in addition to physiotherapists [36].

**Figure 3:**
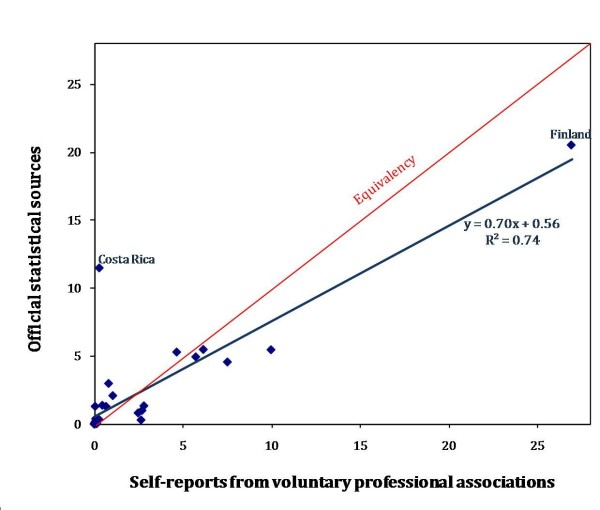
**Density of physiotherapists by data source, 33 countries**. Source: Global Atlas of the Health Workforce; World Confederation for Physical Therapy [19,22]. Note: Countries included are Australia, Bahrain, Bangladesh, Bolivia, Brazil, Cameroon, Canada, Costa Rica, Egypt, Estonia, Finland, Ghana, Indonesia, Iran, Jordan, Kenya, Namibia, Nepal, New Zealand, Nigeria, Panama, Romania, Rwanda, South Africa, Sri Lanka, Swaziland, Tanzania, Thailand, Uganda, United Kingdom, USA, Zambia and Zimbabwe.

While our analysis did not enable us to quantify a "true" value for physiotherapists density, we did find relatively low variability (*R*^*2 *^= 0.74) across the two information sources among the sub-set of countries with comparable data, with densities reported from professional associations tending to be less than official statistics, especially at higher density levels (Figure 3).

### Supply-need relationship

**Figure 4:**
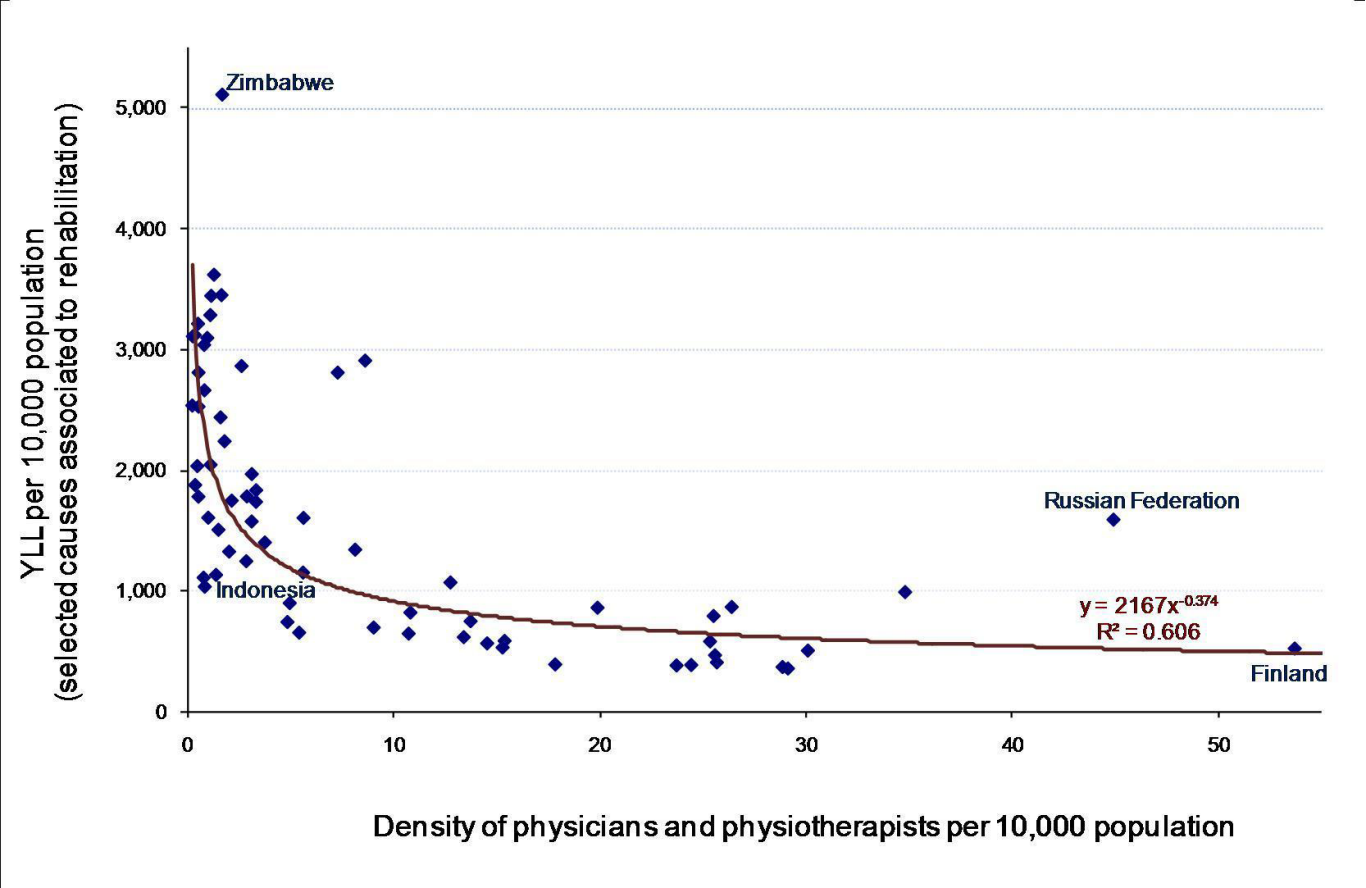
**Attributable years of life lost versus density of health professionals associated to rehabilitation, 67 countries**. Source: Global Atlas of the Health Workforce; GBD Disease and injury country estimates [19,23].

Baseline findings suggest that 92% of the burden of disease in the world (measured in terms of attributable years of life lost, or YLL) is related to causes that require assistance of health professionals associated to rehabilitation (e.g. physiatrists, physical therapists, audiologists, occupational therapists, orthotists, prosthetists, speech-language pathologists and others). A plot of supply of selected categories of health professionals against selected causes of YLL shows a strong and negative relationship, suggesting that countries with the highest burden of disability-related health conditions simultaneously tend to be those with the lowest supply of health workers skilled in rehabilitation services (Figure 4).

**Figure 5:**
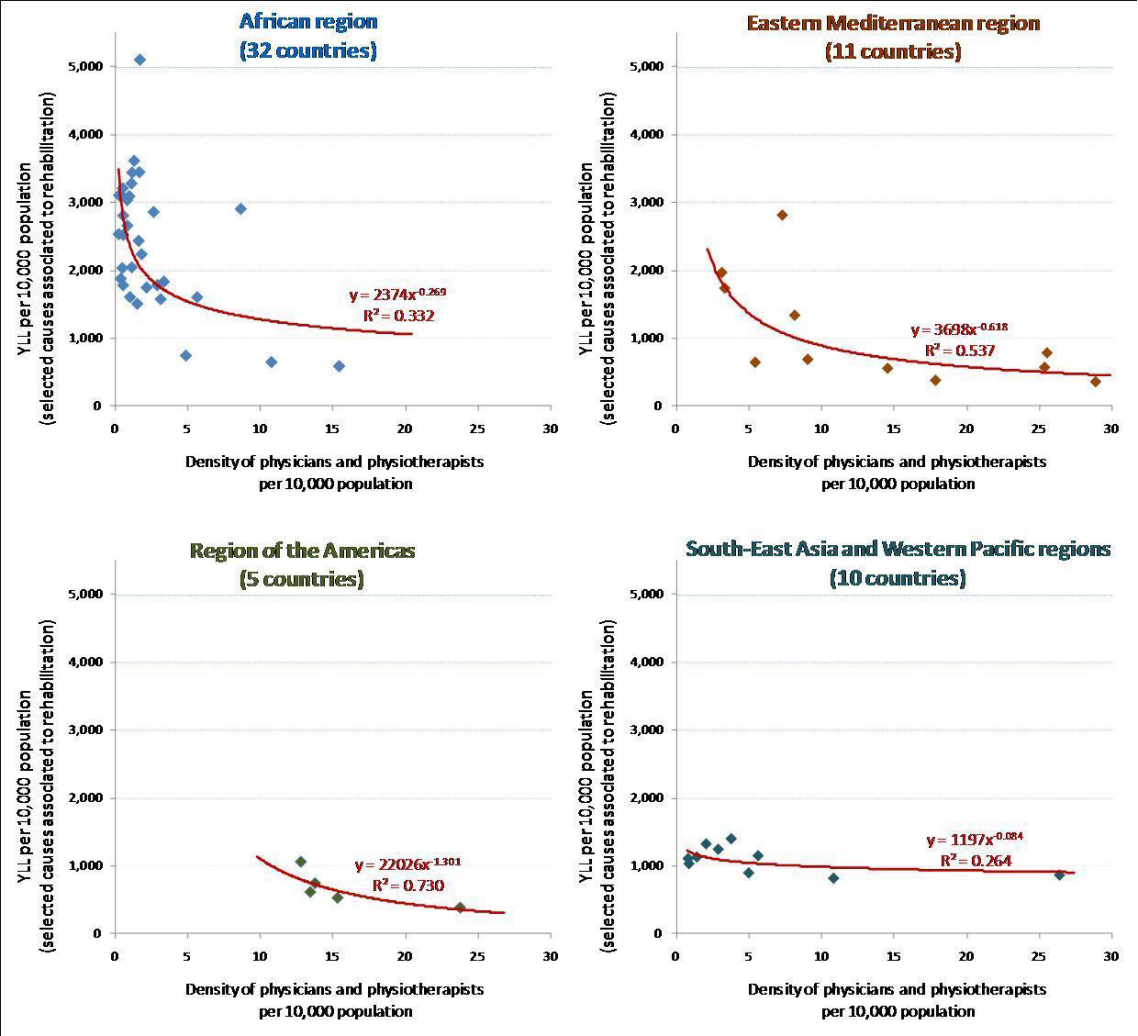
**Attributable years of life lost versus density of rehabilitation health professionals, by region, 58 low- and middle-income countries**. Source: Global Atlas of the Health Workforce; GBD Disease and injury country estimates [19,23]. Note: Country geographical classification based on WHO regions.

Disentangling the analysis by geographical region, a similar pattern emerges among low- and middle-incomes countries (Figure 5). Within regions, countries with higher rehabilitation needs tend to have lower numbers of skilled health workers. At the same time, the fit of the relationship varies across regions: a closer supply-need predictive link in the region of the Americas (*R*^*2 *^= 0.73), less obvious in the South-East Asian/Western Pacific regions (*R*^*2 *^= 0.26).

**Figure 6:**
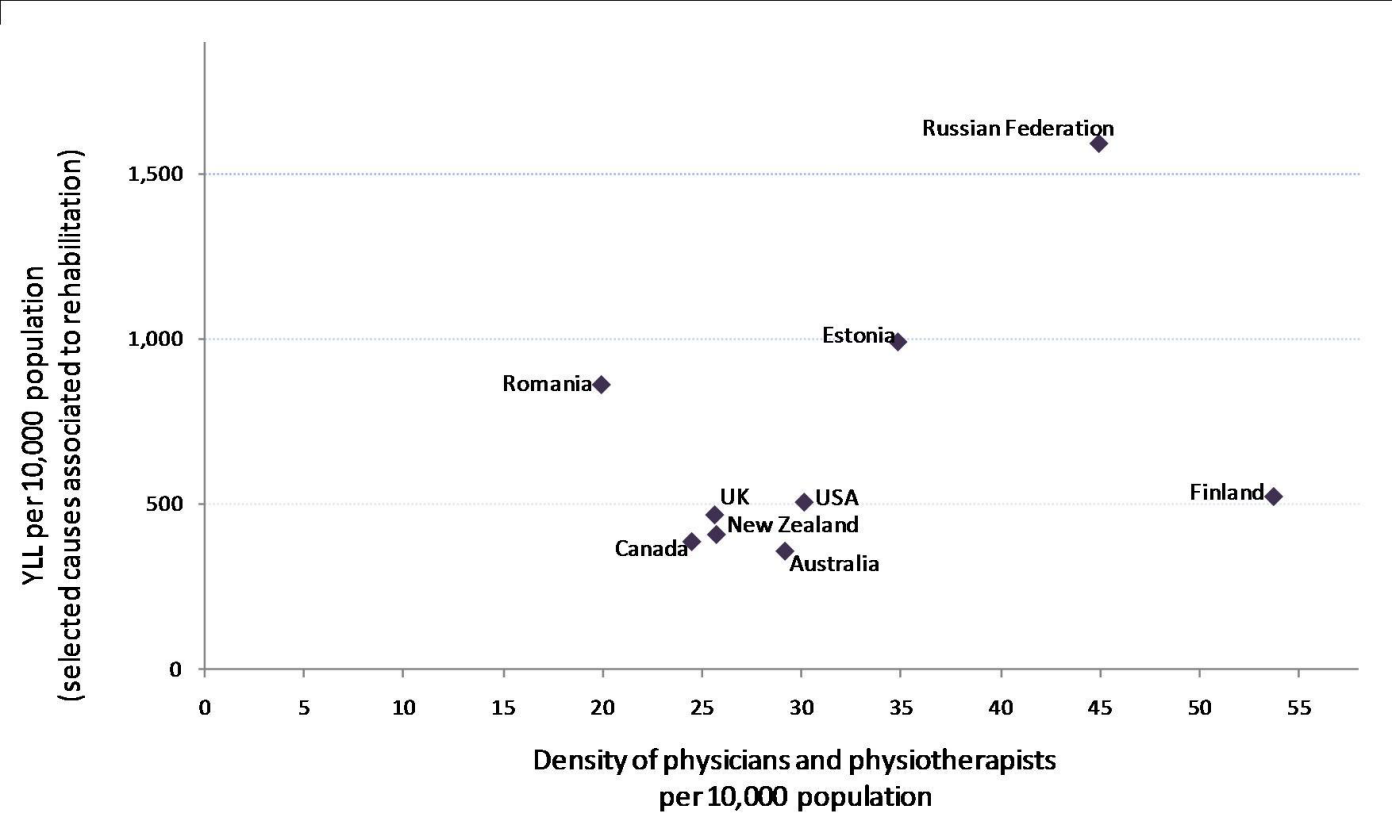
**Attributable years of life lost versus density of rehabilitation health professionals, 9 high and upper-middle income countries**. Source: Global Atlas of the Health Workforce; GBD Disease and injury country estimates [19,23]. Note: Country income classification based on World Bank criterion for classifying national economies.

The picture is more ambiguous among the grouping of high and upper-middle income countries, where there is a lack of a clear supply-need relationship (Figure 6). This grouping includes a heterogeneous collection of countries across the Americas, European and Western Pacific regions, characterized by relatively higher overall levels of HRH supply but varying health system organizations, workforce mixes and disease burdens, especially with regard to the transitional Eastern European countries. However, not counting the latter from the analysis does not necessarily result in a clearer view: no strong monomial relationship is observed among the remaining high income countries with developed market economies, even when excluding the outlier point for Finland (results not shown).

## Conclusions

The findings from this study offer the first global portrait of supply-need dynamics for human resources for rehabilitation. Overall, and sadly not surprisingly, lower supplies of rehabilitation health professionals were found among low- and middle-income countries, including many located in sub-Saharan Africa, where the disease burden related to causes requiring rehabilitation professional skills tends to be greatest. The negative supply-need link was found to generally hold for developing countries, but the strength of the relationship varied across regions of the world. No discernable relationship was teased among the subset of countries with developed and transitional economies with official HRH data available in the public domain.

Given the wide differences observed here across countries and regions in the numbers and distribution of rehabilitation personnel, it remains uncertain whether the currently available data are sufficient to allow analysts and decision makers to draw policy-relevant conclusions. One outstanding challenge in strengthening the global evidence base is the setting of common definitions and classifications of who are rehabilitation health workers. We attempted to enhance cross-national comparability through the mapping of data following internationally standardized classifications for social and economic statistics, notably the International Standard Classification of Occupations for workforce data. However differences in the nature of national economies, health systems and information systems often make it difficult to obtain comparable data. Important discrepancies may be attributed to the number of occupations included in the original information source (and whether health workers' current practice area was actually in rehabilitation services), timeliness of the available data (or lack thereof), and differences in coverage (e.g. whether the source covers health workers in all sectors: public, private, community based services, etc.).

At the same time, health professional density is not necessarily the most important factor in improving population health and welfare. For example, Indonesia and Zimbabwe have similarly low reported numbers of professionals associated to rehabilitation (fewer than 2 physicians and physiotherapists per 10,000 population) but highly divergent disease burdens attributable to associated causes (Figure 4). Both countries have implemented community-based rehabilitation programmes in order to attempt to address service deficiencies in areas with critical shortages of physicians, nurses, and other health and social care professionals [37]. The evidence base is growing for the effectiveness of some non-traditional or alternative cadres in delivering CBR services in lower income settings [38] - although such categories of health workers may not always be adequately captured in national HRH statistics. From a monitoring and evaluation perspective, significant challenges remain in defining and measuring the available workforce with a broader skill set across countries and over time within countries.

International calls are growing for improved collection, analysis and translation of information into evidence that can be used for purposes of HRH policy, planning, programming and accountability [15,39]. This analysis was limited by partial data availability and by heterogeneity in the information sources accessed. In order to monitor trends in health workforce situation and performance, or for countries to share experiences and best practices, it is necessary to know how health workers are defined and classified in the original information source. For example, we found systematic differences in reported supply of physiotherapists according to the nature of the national data source.

Nevertheless, we believe this study provides much needed information on the current global status of human resources for rehabilitation, and hope that it will act as a catalyst for improving the future supply of and demand for quality evidence and research on this topic. For one, it is expected that possibilities for health workforce analyses will be strengthened in the current global series of censuses, known as the 2010 round, which will largely be able to exploit the new ISCO-08 revision [21]. Understanding and strengthening health systems capacity to meet population health and rehabilitation service needs requires better information and evidence on the range of human resources for rehabilitation at the local, national and international levels. All over the world, people with disabilities have many unmet health and rehabilitation needs, yet continue to face important barriers in accessing mainstream health care services and consequently have poorer health outcomes - a double burden in low- and middle-income countries [2]. Improving the availability and use of timely, comprehensive and reliable data on the different health occupations associated to rehabilitation is the first step towards evidence-informed workforce development strategies in rehabilitation.

## Competing interests

The authors declare that they have no competing interests.

## Authors' contributions

NG prepared the first draft of the manuscript. CCL conducted database management and preliminary statistical analysis. MDL contributed to data collection and analysis. All authors participated in the research design, literature review and writing, and read and approved the final version.

## Pre-publication history

The pre-publication history for this paper can be accessed here:

http://www.biomedcentral.com/1472-6963/11/276/prepub
